# Multi-omics and artificial intelligence predict clinical outcomes of immunotherapy in non-small cell lung cancer patients

**DOI:** 10.1007/s10238-024-01324-0

**Published:** 2024-03-30

**Authors:** Ting Mei, Ting Wang, Qinghua Zhou

**Affiliations:** 1 Department of Medical Oncology, Cancer Center, West China Hospital, Sichuan University, Chengdu, Sichuan China; 2 Lung Cancer Center, West China Hospital, Sichuan University, Chengdu, 610000 China

**Keywords:** Immune checkpoint inhibitors, Multi-omics, Artificial intelligence, Immunotherapy, Non-small cell lung cancer

## Abstract

In recent years, various types of immunotherapy, particularly the use of immune checkpoint inhibitors targeting programmed cell death 1 or programmed death ligand 1 (PD-L1), have revolutionized the management and prognosis of non-small cell lung cancer. PD-L1 is frequently used as a biomarker for predicting the likely benefit of immunotherapy for patients. However, some patients receiving immunotherapy have high response rates despite having low levels of PD-L1. Therefore, the identification of this group of patients is extremely important to improve prognosis. The tumor microenvironment contains tumor, stromal, and infiltrating immune cells with its composition differing significantly within tumors, between tumors, and between individuals. The omics approach aims to provide a comprehensive assessment of each patient through high-throughput extracted features, promising a more comprehensive characterization of this complex ecosystem. However, features identified by high-throughput methods are complex and present analytical challenges to clinicians and data scientists. It is thus feasible that artificial intelligence could assist in the identification of features that are beyond human discernment as well as in the performance of repetitive tasks. In this paper, we review the prediction of immunotherapy efficacy by different biomarkers (genomic, transcriptomic, proteomic, microbiomic, and radiomic), together with the use of artificial intelligence and the challenges and future directions of these fields.

## Introduction

Lung cancer is responsible for the majority of cancer-related deaths in the world. Non-small cell lung cancer (NSCLC) represents approximately 80–90% of lung cancers [1]. Over half of NSCLC tumors are advanced when diagnosed, and the overall 5-year survival for NSCLC patients is only 18% [2].

In recent years, the use of various types of immunotherapy, particularly immune checkpoint inhibitors (ICIs) targeting programmed cell death 1 (PD-1) or programmed death ligand 1 (PD-L1), has revolutionized the treatment of NSCLC [3, 4]. Both PD-1 and PD-L1 are important immune checkpoints (ICs). PD-1 is expressed on the surfaces of immune cells and binds its ligand, PD-L1, expressed on tumor cells, suppressing the immune response against the tumor [5]. The function of anti-PD-1/PD-L1 immunotherapy is to prevent this interaction and thus enhance the immune response directed against the tumor [6].

Immunohistochemical (IHC) detection of PD-L1 has FDA approval and is the most frequently used biomarker for the prediction of the effectiveness of immunotherapy [7]. However, anomalous results have been observed in clinical practice, with many patients with high PD-L1 levels showing a poor response to immunotherapy while others with low or negative PD-L1 levels do respond. Furthermore, the efficacy of immunotherapy guided by PD-L1 expression levels does not exceed 30% [8]. This suggests that the use of PD-L1 as a sole biomarker is inadequate.

The tumor microenvironment (TME) contains various cell types, including tumor, stromal, and infiltrating immune cells, and its composition differs significantly within tumors [9], between tumor types [10], and among individuals [11]. In such a heterogeneous system, many pro- and anti-tumor cellular components or signals can influence the efficacy of immunotherapy. Thus, the tumor response to immunotherapy is complex and involves a large number of mechanisms and pathways. Omics approaches, aimed at the comprehensive assessment of each patient through the analysis of high-throughput-extracted features, promise to provide a more comprehensive characterization of this complex ecosystem.

Artificial intelligence (AI) is a subdivision of computer science that can be used to predict and classify material according to the available data [12]. Deep learning (DL) is a representative learning method in which complex multi-layer neural network architectures automatically learn from data by converting input information into multi-level abstractions. At the same time, models are constructed by the selection of features and model fitting using a combination of computerized algorithms and high-throughput data. Previous studies have shown that AI-based automatic learning and diagnostic models have shown good performance in cell classification [13], cancer detection [14], pathological diagnosis [15], and the analysis of multiple biomarkers [16].

**Fig.1:**
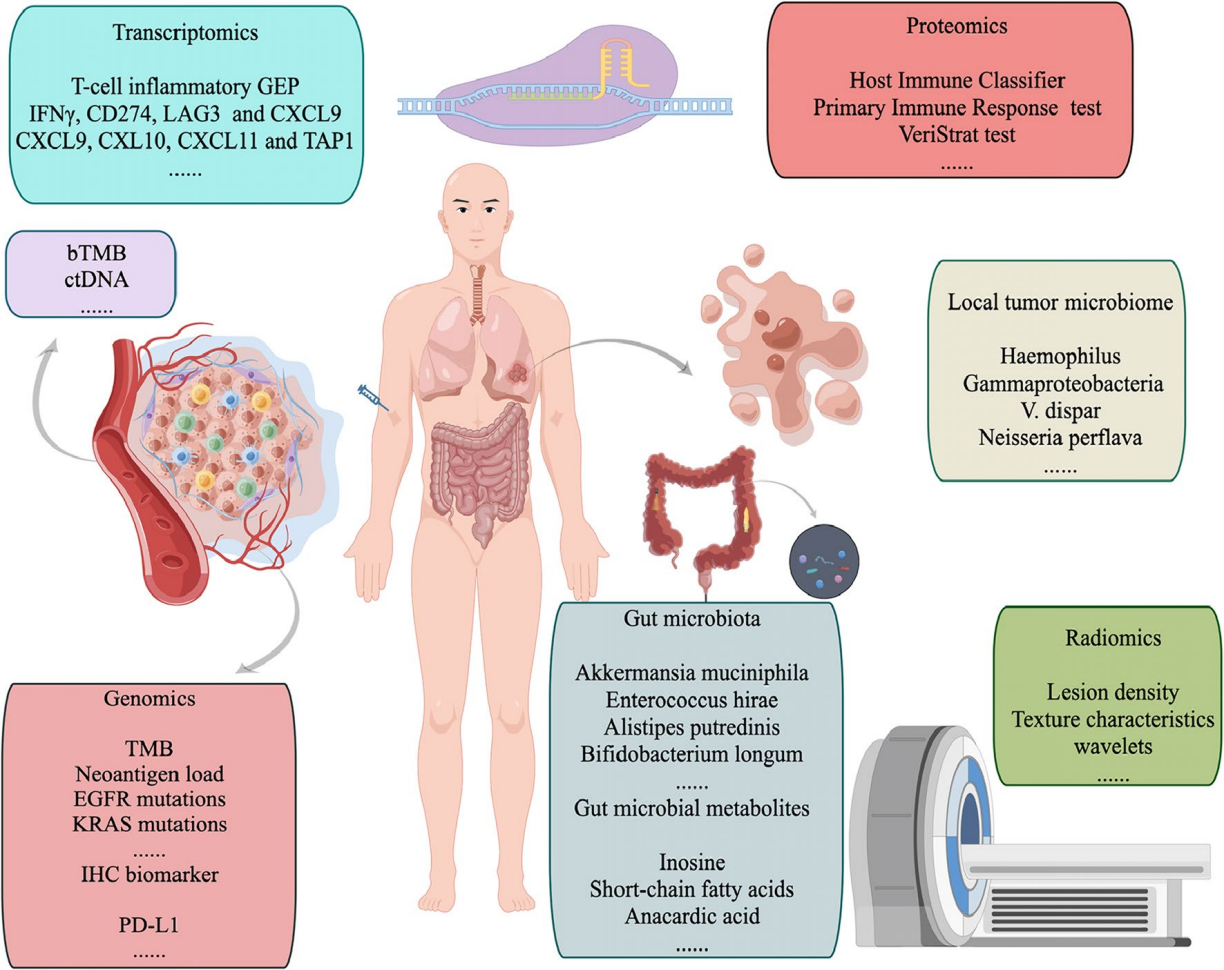
Overview of multi-omic signatures for predicting the efficacy of immunotherapy in patients with non-small cell lung cancer

Here, we review the use of multi-omics (genomics, transcriptomics, proteomics, microbiomics, and radiomics) for the prediction of the outcome of immunotherapy and specifically discuss the applications of AI in these predictions (Fig. 1).

## Genomics

### Tumor mutation burden

The TMB represents the total number of somatic mutations present in the tumor tissue after the exclusion of germline mutations. Somatic mutations can produce changes in cell surface proteins, leading to the appearance of neoantigens that are recognized as foreign by the immune system, stimulating the immune response and subsequent elimination of the tumor cells [17]. There is, thus, a significant association between high TMB and the patient’s response to immunotherapy.

In the CheckMate 026 study, an exploratory analysis of the influence of tumor mutational burden (TMB) on clinical outcome showed that in patients with high TMB levels (≥ 243 mutations/Mb), those receiving immunotherapy had longer PFS (9.7 months vs. 5.8 months for chemotherapy, HR, 0.62 (95%CI, 0.38–1.00)) and higher objective response rates (ORRs) (46.8% vs. 28.3% for chemotherapy) [18]. The Checkmate 227 study used TMB ≥ 10 mut/Mb as the cutoff value for high TMB and found markedly improved ORR and PFS when nivolumab was combined with Ipilimumab compared with chemotherapy in advanced NSCLC patients with high TMB, regardless of PD-L1 expression level [19]. These two studies suggest that the TMB may be useful as a complementary biomarker for the selection of patients likely to respond to immunotherapy. Next, the bFAST cohort C study and a multi-center retrospective study set the TMB threshold at 16 mutations/Mb and 20 mutations/Mb, respectively, and also found that high TMB was associated with better patient outcomes [20, 21]. However, there is a problem that these studies have inconsistent definitions of high TMB, and the inconsistencies in TMB detection platforms and methods, sample status, and result interpretation further interfere with the prediction of clinical outcomes. Therefore, TMB requires synchronization and standardization for reliable clinical application. In addition, it is worth noting that TMB still has serious limitations as a predictive factor in immunotherapy combined with chemotherapy.

### Circulating tumor DNA (ctDNA)

Measurement of ctDNA in the peripheral blood can provide an accurate reflection of the TMB and genomics of the cancer and can thus be used as a biomarker predicting treatment response and prognosis.

Assessment of ctDNA levels before treatment has been demonstrated to be useful for outcome prediction, with high ctDNA scores indicative of poor outcomes in many cancer types [22, 23]. A prospective phase II clinical trial observed an association between lower baseline ctDNA levels and enhanced OS and PFS after treatment of NSCLC with pembrolizumab [24]. Several recent studies have shown an independent association between early reduction in the ctDNA allele frequency and enhanced OS and ORR in patients with advanced NSCLC after immunotherapy [24, 25]. Vokes et al. reported at the 2023 American Society of Clinical Oncology (ASCO) that dynamic changes in ctDNA can predict the therapeutic efficacy of cemiplimab [26]. Pseudoprogression can also be identified by ctDNA kinetics. For instance, a study by Guibert et al. observed rapid and significantly reduced ctDNA levels in two patients with pseudoprogression while patients with true progression showed increased levels [27].

Although ctDNA levels are considered predictors of response to immunotherapy, there is no uniform standard for baseline risk stratification thresholds and time points for testing. Therefore, further cross-study validations and standardization of ctDNA thresholds are needed in the future.

### Genetic mutations

#### EGFR mutations

Together with the total mutational burden, there are specific mutations that may influence the efficacy of immunotherapy. Meta-analyses of three clinical trials (CheckMate 057, POPLAR, and KEYNOTE 010) and meta-analyses of five trials (CheckMate 017, CheckMate 057, KEYNOTE 010, OAK, and POPLAR) confirmed that single-agent ICI does not prolong OS in patients with mutations in EGFR [28, 29]. The CheckMate 012 study found that immunotherapy combined with chemotherapy similarly failed to improve PFS and OS in patients with EGFR mutations [30]. However, the ORIENT-31 study confirmed that ICI combined with anti-angiogenic therapy and chemotherapy significantly improved PFS in EGFR-mutant non-squamous NSCLC patients who progressed after treatment with EGFR-TKIs [31]. Although the IMpower150 [32], ORIENT-31 and ATTLAS/KCSG-LU19-04 [33] studies have unanimously confirmed that patients with EGFR-TKI-resistant NSCLC can benefit from the four-drug combination therapy of immune + platinum-containing double-drug chemotherapy + bevacizumab. However, the four-drug combination regimen has a higher incidence of adverse effects and is limited in clinical use. Checkmate 722 and Keynote 789 studies chose immunotherapy + platinum-based doublet chemotherapy, but the efficacy was not satisfactory. The Checkmate 722 study demonstrated that nivolumab + chemotherapy had no significant clinical benefit compared with chemotherapy in patients with EGFR-TKI resistance [34]. In the Keynote 789 study, pembrolizumab repeated the failed results [35]. Whether other immune drugs combined with chemotherapy are effective, and whether immunotherapy + anti-angiogenic therapy + single-agent chemotherapy is feasible and is worthy of further exploration in the future.

#### ALK or ROS1 fusion-positive

NSCLC patients with the ALK/ROS1 fusion are classified as having low TMB despite their high levels of PD-L1 expression [36]. The ATLANTIC study has shown that the ORR of patients with ALK rearrangements after immunotherapy was significantly lower than that of ALK-negative patients [37]. The results of the IMMUNOTARGET study found that the ORRs of NSCLC patients with positive ALK and ROS1 driver genes treated with ICIs were 0% and 17%, respectively [38].

#### RET rearrangement

Patients with NSCLC accompanied by RET rearrangements also typically have lower TMB levels (< 2.5 mut/Mb) and PD-L1 expression [36, 39]. Previous retrospective studies have shown that the curative effect of ICI therapy in patients with RET-rearranged NSCLC is poor, with a median PFS of < 3.5 months and an ORR of < 10% [40, 41].

#### HER2 mutations

Marcelo et al. analyzed data from more than 4000 driver-positive NSCLC patients, observing that patients with classic EGFR mutation and HER2 mutations who were treated with ICIs had the shortest PFS (1.8 and 1.9 months, respectively) [36]. The POLISH study also found that for advanced NSCLC patients with HER2 mutations, chemotherapy + immunotherapy did not improve median PFS compared with chemotherapy + anti-angiogenic therapy or chemotherapy alone (both *p* > 0.05) [42].

#### KRAS mutations

Mutations in KRAS are often associated with both a high TMB and significantly elevated PD-L1 expression [43]. Moreover, previous studies found no significant differences in ORR, PFS, and OS between NSCLC patients with or without KRAS mutations [44, 45].

Mutations in KRAS are highly heterogeneous. Ricciuti et al. found that among patients with KRAS mutations receiving immunotherapy, those with the KRAS G12D mutation had worse ORR, PFS, and OS than patients with other KRAS mutations [46].

KRAS mutations in NSCLC are frequently seen in conjunction with mutations in tumor protein 53 (TP53) and serine-threonine kinase 11/liver kinase B1 (STK11/LKB1). These KRAS-STK11/LKB1 co-mutant tumors tended to show greater resistance to PD-1 inhibitors [47], while the patients with the KRAS-TP53 co-mutation were more sensitive to PD-1 inhibitors [48]. Lung cancer patients with KRAS mutations have a 20% and 10% chance of co-occurring KEAP1 and SMARCA4 mutations, respectively [49, 50], and these co-mutations appear to be linked to poorer outcomes after immunotherapy.

#### BRAF mutations

BRAF-mutant NSCLC often has a high TMB and high levels of PD-L1 expression [36, 51]. Li et al. found no significant difference in PFS, OS, and ORR between NSCLC patients carrying BRAF-mutant or wild-type genes after single-agent ICI therapy or ICI combination therapy [52]. A study observed shorter PFS and OS in patients receiving ICIs in the patients carrying the BRAF V600E mutation compared with those without the mutation [53].

#### MET exon 14

Studies by Negrao et al. and Mazieres et al. found that NSCLC patients with MET exon 14 mutations who were treated with ICIs had a median PFS of < 5 months and an ORR of < 20% [36, 38]. Similarly, Sabari et al. found that the overall response to ICIs was reduced in NSCLC patients with MET exon 14 mutations regardless of PD-L1 expression and the TMB level [54].

## Transcriptomics

Transcriptomics involves the analysis of all RNA transcripts within a cell type and is usually performed using high-throughput techniques such as RNA sequencing (RNA-seq) and microarrays [55]. A number of recent studies have investigated the prediction of immunotherapy response using transcriptome signatures.

Ayers analyzed gene expression profiling (GEP) using RNA from baseline tumor samples from patients treated with pembrolizumab, identifying the GEPs of T-cell inflammation, specifically, genes involved in antigen presentation after IFN-γ activation, the expression of chemokines, cytotoxicity, and resistance to the adaptive immune response [56]. Another clinical trial involving 20 groups of patients with advanced solid tumors further validated the relationship between T-cell inflammatory GEP and the clinical efficacy of pembrolizumab and found that tumors with higher T-cell inflammatory GEP showed higher response rates and longer PFS [57]. Further analysis by the POPLAR study showed that among patients treated with atezolizumab, patients with high expression of T effector-interferon-γ-related genes had significantly longer OS [58]. In addition, in NSCLC patients receiving durvalumab, those with a four-gene IFNγ-positive (IFNγ +) signature, namely IFNγ, CD274, LAG3, and CXCL9, showed greater ORR and longer median OS and PFS, irrespective of their PD-L1 status [59]. These transcriptomic data can help to further optimize cancer immunotherapy strategies. However, these studies have tended to analyze specific genes in isolation, and the underlying pathways through which the genes influence the efficacy of ICI therapy require further investigation.

## Proteomics

Protein is one of the main building blocks of cells. Proteomics is the large-scale study of proteins through analysis of their identities and quantities in biological samples (cells, tissues, or body fluids).

Using spatially resolved proteomic analysis, Myrto et al. identified tumor cell CD44 as a biomarker for the prediction of the efficacy of immunotherapy in NSCLC patients [60]. Shen et al. analyzed SEL1L3 in a large-scale tissue proteomics dataset established by the Taiwan Cancer Moon Shot Program and found that it could serve as a potential emerging adjuvant for immunotherapy of lung adenocarcinoma [61]. To verify the real performance of blood-based proteomic analysis in NSCLC immunotherapy, all patients were tested and designated as HIC-Hot (HIC-H) or HIC-Cold (HIC-C) before initiating treatment. The study found that the survival time of all HIC-H patients was significantly longer than that of HIC-C patients, irrespective of PD-L1 expression. Additionally, the data also suggest that HIC-C patients should not be treated with ICIs alone, regardless of PD-L1 expression [62]. Furthermore, the combination of protein markers (CXCL9, CXCL10, IL-15, CASP8, and ADA) was found to be more accurate in predicting response than tumor PD-L1 expression or the levels of individual proteins [63].

Notably, although proteomic studies are able to predict the immunotherapy response, there are still some limitations. These tests are based on protein signatures, and the quality of the signature is determined by the quality of the data used in the signature generation. In addition, proteomic analyses based on tumor blood typically measure proteins shed from the tumor into the blood. Thus, the effectiveness of these tests is dependent on the amount of shedding, which is likely to vary between patients. Finally, most studies focusing on proteomics-based biomarkers have been retrospective, and as such they lack informed treatment guidelines, such as NCCN guidelines. Thus, more prospective and randomized clinical trials are required in this area.

## Microbiomics

The microbiome refers to the colonies of symbiotic microorganisms within the human body. Analysis of the microbiome is also thought to be able to predict the response to ICI treatment.

### Gut microbiota

A study published by Routy in 2018 showed that antibiotic treatment reduced the OS of patients with advanced NSCLC by half within 3 months of immunotherapy [64]. Significant differences in fecal microbiomes were observed before the start of ICI treatment between patients who responded to treatment and those who did not, with *Akkermansia muciniphila* and *Enterococcus hirae* found to be significantly related to improved clinical outcomes. Routy et al. reported that ICI resistance could be reduced in mice by transplantation of *Akkermansia muciniphila* and *Enterococcus hirae*, suggesting that the gut microbiome modulates the effectiveness of immunotherapy and that ICI efficacy is related to gut bacterial type and can improve immune tolerance in patients by supplementing with beneficial bacteria. A multi-center prospective observational study in 2019 further verified that among NSCLC patients treated with ICI, those with higher levels of *Akkermansia muciniphila* showed better response rates [65].

These studies demonstrated that the microbiome composition differs according to treatment response, and while favorable results were consistently associated with increased microbiome diversity, differences were observed in the individual bacteria between studies. These differences may be due to the type of ICI used, and geographical location may also play a role.

### Local tumor microbiome

The gut microbiota has been linked to the therapeutic effectiveness of immunotherapy through a variety of mechanisms. For lung cancer, local tumor-microbiome interactions are also crucial for the efficacy and prognosis of ICI therapy. The results of Brutsche et al. showed that, as with gut microbes, more diverse bacterial metagenomes were linked to improved survival after ICI treatment. The presence of *Gammaproteobacteria* has been associated with reduced PD-L1 levels and decreased response to immunotherapy [66]. Sang Hoon Lee et al. observed that *V. dispar* was more abundant in patients that responded to immunotherapy while *Haemophilus influenzae* and *Neisseria perflava* were more abundant in patients who did not. Thus, *Haemophilus* may represent a target for lung cancer treatment [67]. However, mechanistic data on the function of the microbiome in relation to NSCLC immunotherapy are scarce at this stage, and further investigation is required.

Both preclinical and clinical studies have provided insight into the influence of the microbiome on the immunotherapy response. Future studies should address the classification of favorable and unfavorable characteristics of the microbiota and their influence on the cells and pathways of the immune response to provide a full understanding of their roles in TMEs and to enhance the effectiveness of treatment. The gut-pulmonary axis refers to the commonality between the microbiomes of the lung and gut which are not separate entities but rather communicate in a bidirectional manner to modulate each other's immune responses. The gut-pulmonary axis offers the possibility of indirect modulation of the lung microbiota by manipulating the more accessible gut microbiota.

## Radiomics

Radiomics converts medical images into quantitative data, dissecting the complexity of tumor biology both macroscopically and microscopically. Radiomics offers a more comprehensive analysis of the tumor than analysis of biopsy specimens as it extracts features from the entire TME. Radiomic models can also be applied in multiple scans, allowing the non-invasive and continuous monitoring of the tumor and its response to treatment.

### Prediction of immunotherapy efficacy

Tunali et al. analyzed pre-immunotherapy data from NSCLC patients treated with PD-1/PD-L1 from 13 institutions, finally identifying four radiomic features that were used for the construction of a model for the differentiation of patient response to ICI with an AUC of 0.790 [68]. Lu et al. selected 88 radiomic features from CT images to build a random forest model and combined clinicopathological factors to determine the patients that were most likely to respond to immunotherapy before beginning treatment (AUC: 0.848–0.902) [69]. Using a combination of CT images and genomic data, Sun et al. developed a radiomic signature of CD8 cells that were effective in predicting treatment outcomes with anti-PD-1 or anti-PD-L1 immunotherapy [70].

### Identification of hyperprogression and pseudoprogression

Hyperprogression describes the apparent paradox of increased progression occurring after the initiation of immunotherapy. Patients who experience hyperprogression have a poor prognosis, and early identification of hyperprogression may prevent ineffective treatment and reduce the occurrence of toxicity. Pranjal Vaidya et al. extracted a total of 198 radiomic texture patterns as well as nodule-associated vascular features from CT scans for modeling to identify hyperprogression, demonstrating AUCs of 0.850 and 0.960 for the training and validation sets, respectively [71]. In addition, Tunali et al. combined radiomic models with clinical features and found relatively high predictive power for hyperprogression, with AUCs ranging from 0.804 to 0.865 [72]. In addition, distinguishing pseudoprogression from true disease progression is important for the clinical decision-making process. Barabino et al. found that changes in nine radiomic features were significantly associated with pseudoprogression [73].

These studies show that while radiomics can be a better predictor of ICI treatment efficacy, several problems remain, including a lack of high-quality data and image standardization, together with a poorly effective combination of multiple imaging methods, and a lack of prospective validation. In future studies, a variety of modeling approaches should be used for the selection of the best method. Furthermore, multiple cross-validations should be conducted for single-center studies. In conclusion, we consider that the use of radiomics in PD-L1-related imaging of NSCLC patients has potential.

## The application of AI in predicting the response to immunotherapy

### AI-based genomics

He et al. used 1020 DL features to build a model to distinguish between patients with high and low TMB, finding that the model was effective in dividing patients into high-risk and low-risk groups when predicting the efficacy of immunotherapy [74]. Jain and Massoud combined three DL models to predict TMB status, reporting an AUC of the joint model of 0.920 [75]. These studies demonstrate the value of an AI system to assist healthcare professionals in identifying the expression status of these genes that have the advantages of being easy to use and non-invasive.

### AI-based transcriptomics

Intratumoral heterogeneity (ITH) is a key factor influencing the patient response to immunotherapy [76]. To determine ITH levels, Sung et al. used an ML-based approach to predict ITH using transcriptomic data. They demonstrated that their method could distinguish tumor samples with high and low ITH levels and identify transcriptomic markers associated with ITH [77]. To facilitate the investigation of heterogeneity in the ICI response, Zeng et al. developed a non-negative matrix factorization (NMF)-based ML framework to identify factors affecting the immunotherapy response using data from TCGA samples [78]. With the advent of the era of precision medicine, AI-assisted transcriptomic technology will continue to develop and become more common in immunotherapy research, providing valuable information for the formulation of new strategies to improve the efficacy of cancer immunotherapy.

### AI-based proteomics

A study reported the construction of an ML-based serum protein classifier that could classify patients into drug-resistant, intermediate, or sensitive groups according to clinical and mass spectrometry (MS) characteristics. This serum protein classifier showed good performance in predicting patient immune responses [79]. The VeriStrat test is a proteomic signature using ML-based MS. The test evaluates the spectrum of blood samples and assigns patients to either VeriStrat "good" (VS-G) or VeriStrat "poor" (VS-P) groups. It was found that the VeriStrat status was significantly associated with both PFS and OS in NSCLC patients receiving ICI treatment [80].

Compared to genomics and transcriptomics, proteomics may be more effective in assessing features of the TME and immune responses in patients with lung cancer, resulting in a more accurate prediction of the response to immunotherapy. The application of AI methods to proteomics may thus represent a future direction in the development of improved methods for assessing patient prognosis after immunotherapy.

### AI-based microbiomics

Many factors associated with the gut microbiota, including its composition and community structures, influence the immunotherapy response. Thus, statistical analysis using AI can assist in the elucidation of the specific composition or important combinations of gut microbiota species. Tatsuro Okamoto will conduct a prospective observational study using AI (UMIN000046428) to elucidate the specific gut microbiome composition or gut microbial combinations associated with the immunotherapy response in lung cancer patients [81].

### AI-based radiomics

Based on contrast-enhanced CT before neoadjuvant immunotherapy, a study used radiomic features, clinicopathological information, and DL features to construct a model for the prediction of a good pathological response, finding an AUC of 0.805 [82]. In another study, a combination of radiomics and DL was used for predicting the response of NSCLC patients with advanced disease to immunotherapy with an AUC of 0.960 [83]. He et al. also demonstrated that the combination of radiomics with DL could identify patients likely to benefit from ICI treatment (*p* < 0.001) [84].

Radiomics is both non-invasive and reproducible and thus offers a novel solution. Also, radiomics-based predictive models allow the early identification of suitable patients for immunotherapy, thus enabling precision medicine.

## Future challenges

Although various studies have explored the potential of various histological approaches and AI in predicting the response to immunotherapy, challenges associated with their application in clinical practice still remain.

Both the methods of measurement and the platforms used to measure most genomic biomarkers are inconsistent, resulting in poor reliability and reduced accuracy. Furthermore, the optimal threshold is difficult to determine, i.e., different studies have used different thresholds for prediction, leading to inconsistent results. MS-based proteomics has been widely used in cancer immunology research, but MS proteomics has difficulty in accurately identifying unique peptides and different protein isoforms. Transcriptomics also presents several difficulties, specifically, in relation to sample preparation, computational analysis, and reproducibility. As for microbiomics, its future challenges include an inadequate understanding of the modulating effects of the microbiota on the therapeutic response, a lack of information and consensus on the use of microbial signatures as predictive biomarkers, and limited information on how the microbiota could be modulated. In addition, while current research has focused on bacteria, symbiotic viruses, fungi, and archaea also have non-negligible roles in cancer. In terms of radiomics, a significant limitation is the absence of standardization between studies as this complicates data sharing and reduces the generalizability of models constructed in different institutions (Table 1).

**Table 1:** Advantages and disadvantages of biomarkers for predicting immunotherapy outcome

Category	Advantage	Disadvantages
PD-L1	1. Easy to detect 2. FDA-approved	1. Inconsistent detection methods 2. Uncertain optimal cutoff value 3. PD-L1 dynamic changes
TMB	1. FDA-approved 2. Independent of other predictors such as PD-L1 expression	1. Tumor spatiotemporal heterogeneity 2. Inconsistent detection methods 3. Uncertain optimal cutoff value
ctDNA	1. Minimally invasive 2. High repeatability	1.Detection technology and standardization issues 2. ctDNA source and decomposition issues
NAL	1. Improve the accuracy of treatment	1. Technical limitations 2. High data quality requirements 3. Difficulty obtaining data
Gene mutation	1. Wide clinical application	1. Inconsistent detection platform and analysis method 2. Uncertainty in predictions 3. Tumor heterogeneity and dynamics
Transcriptome	1. Aid clinical decision-making 2. Overall	1. Difficult data analysis 2. Uncertainty about data sources 3. Lack of uniform standards
Proteome	1. Minimally invasive (usually only plasma)	1. Protein marker diversity 2. Technical complexity 3. Difficulty in interpreting data
Microbiome	1. Easy access to samples 2. High repeatability	1. Lack of standardized methods 2. Unclear mechanism
Radiomics	1. Non-invasive 2.Multi-parameter analysis 3. High precision 4. Fast	1. Sample size limitation 2. Difficult data analysis 3. High cost

Faced with the challenge of lack of standardization and validation of biomarkers for predicting the efficacy of immunotherapy in NSCLC, the following are some suggestions and directions for future efforts: 1) Standardize measurement methods of biomarkers: Develop unified measurement methods and technical standards to ensure the reliability and comparability of biomarker measurement results. Establish standard operating procedures and quality control procedures to reduce measurement errors. 2) Multi-center collaborative research: Establish an international or cross-institutional research collaboration organization to conduct large-scale research, collect more sample data, and conduct verification and comparative analysis of different immunotherapy drugs and treatment options. Cross-center collaboration can increase the number of samples and improve the credibility of the study. 3) Unified data collection and sharing: Establish a unified database for collecting and storing biomarker data generated in clinical trials and real-world applications. By sharing data, the verification and promotion of biomarkers can be accelerated, and more data can be provided for the discovery and verification of future biomarkers.

The cost-effectiveness of different biomarkers also deserves discussion. Because genomics and radiomics can use existing gene sequencing data and existing medical imaging data, their application in efficacy prediction is relatively low-cost. Microbiomics is less expensive but requires steps such as sample collection and high-throughput sequencing. Transcriptomics and proteomics involve multiple steps such as sample collection, RNA (protein) extraction, sequencing, and analysis, so their costs are relatively high. In low-resource settings, several strategies can help improve the cost-effectiveness: 1) Optimize sample collection and processing procedures to ensure high-quality data acquisition. 2) Reasonably use existing data resources and conduct secondary analysis to reduce the cost of new data collection. 3) Establish cross-institutional or international collaboration networks to share data and resources. 4) Develop simpler, cost-effective measurement technologies, such as rapid sequencing, portable imaging equipment, etc. 5) Further strengthen the development of artificial intelligence algorithms and improve the accuracy and efficiency of prediction models, thereby saving the cost of data analysis and interpretation.

## Conclusions

In this study, we reviewed the application of genomics, transcriptomics, proteomics, microbiomics, and radiomics in predicting immunotherapy outcomes. While data on the use of individual biomarkers to predict response to immunotherapy are plentiful, direct comparisons between them are scarce. PD-L1 is still the most commonly used, and its combination with other biomarkers can help improve the predictive ability of immunotherapy efficacy. In addition, the combination of AI and multi-omics data can help integrate the various data and realize automatic prediction, ultimately providing personalized treatment for patients with lung cancer.

## Data Availability

The data that support the findings of this study are available from the corresponding author upon reasonable request.
